# Dichlorido[2-diphenyl­phosphanyl-*N*-(pyridin-3-ylmeth­yl)benzyl­idenamine-κ^2^ *P*,*N*]platinum(II)

**DOI:** 10.1107/S1600536811040086

**Published:** 2011-10-05

**Authors:** Haleden Chiririwa, Reinout Meijboom

**Affiliations:** a Research Centre for Synthesis and Catalysis, Department of Chemistry, University of Johannesburg, PO Box 524 Auckland Park, Johannesburg 2006, South Africa

## Abstract

The title compound, [PtCl_2_(C_25_H_21_N_2_P)], is a Pt^II^ complex with an NPCl_2_ coordination sphere in which the metal is coordinated to the imino N and phosphane P atoms of the ligand and to two chloride ions. The Pt^II^ atom is in a distorted square-planar environment and is bound to the ligand *via* the P and amine N atoms in a *cis* fashion, with the chlorine atoms located at the two remaining sites.

## Related literature

For related structures with related ligands, see: Chiririwa *et al.* (2011 ▶); Ghilardi *et al.* (1992 ▶); Sanchez *et al.* (1998 ▶, 2001 ▶). For Pt—N and Pt—P bond lengths in imino­phosphane platinum(II) complexes, see: Ankersmit *et al.* (1996 ▶). 
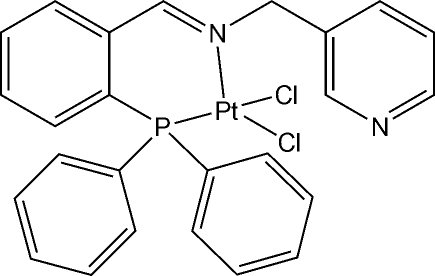


## Experimental

### 

#### Crystal data

[PtCl_2_(C_25_H_21_N_2_P)]
*M* *_r_* = 646.40
Triclinic, 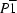

*a* = 9.9684 (14) Å
*b* = 10.4129 (15) Å
*c* = 12.526 (3) Å
α = 97.687 (5)°
β = 98.363 (5)°
γ = 114.499 (3)°
*V* = 1143.1 (4) Å^3^
*Z* = 2
Mo *K*α radiation
μ = 6.46 mm^−1^
*T* = 173 K
0.07 × 0.06 × 0.04 mm

#### Data collection

Bruker Kappa DUO APEXII diffractometer
Absorption correction: multi-scan (*SADABS*; Bruker, 2007 ▶) *T*_min_ = 0.671, *T*_max_ = 0.802
16090 measured reflections
4994 independent reflections
4177 reflections with *I* > 2σ(*I*)
*R*_int_ = 0.058

#### Refinement

*R*[*F* ^2^ > 2σ(*F* ^2^)] = 0.032
*wR*(*F* ^2^) = 0.062
*S* = 1.01
4994 reflections
280 parameters
H-atom parameters constrained
Δρ_max_ = 0.87 e Å^−3^
Δρ_min_ = −1.02 e Å^−3^

### 

Data collection: *APEX2* (Bruker, 2007 ▶); cell refinement: *SAINT* (Bruker, 2007 ▶); data reduction: *SAINT*; program(s) used to solve structure: *SHELXS97* (Sheldrick, 2008 ▶); program(s) used to refine structure: *SHELXL97* (Sheldrick, 2008 ▶); molecular graphics: *X-SEED* (Barbour, 2001 ▶); software used to prepare material for publication: *SHELXL97*.

## Supplementary Material

Crystal structure: contains datablock(s) global, I. DOI: 10.1107/S1600536811040086/go2028sup1.cif

Structure factors: contains datablock(s) I. DOI: 10.1107/S1600536811040086/go2028Isup2.hkl

Additional supplementary materials:  crystallographic information; 3D view; checkCIF report

## Figures and Tables

**Table 1:** Selected bond lengths (Å)

Pt1—N1	2.040 (4)
Pt1—P1	2.1999 (13)
Pt1—Cl2	2.2840 (12)
Pt1—Cl1	2.3806 (14)

**Fig. 1.**
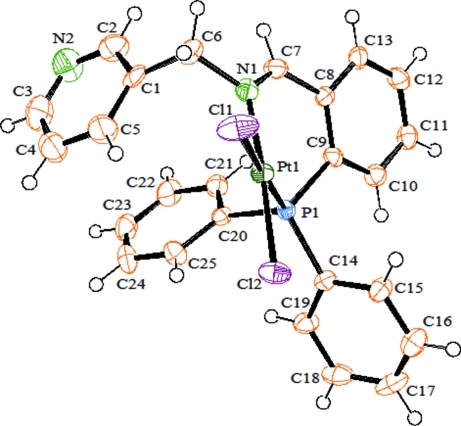
View of (I) (50% probability displacement ellipsoids).

## supplementary crystallographic information

### Comment

In recent years, platinum complexes with iminophosphane ligands of the *N*-[(2-diphenylphosphanyl)benzylidene]amine type have been used as catalysts (or catalyst precursors) in a variety organic reactions. To the best of our knowledge, no structures have been determined so far, concerning the free ligand -(2(diphenylphosphanyl) benzylidene) (phenyl) methanamine, where the potentially bidentate ligand is chelated to the metal through the phosphorus and imino nitrogen atoms (Fig. 1). The platinum is in a square-planar environment and it is bound to the ligand using a k^2^*P*,*N* interaction in a *cis* fashion, with the chlorides located at the two remaining sites. However the square-planar geometry of the platinum environment is distorted with the angles being less than 180°, N(1)-Pt(1)-Cl(2) and P(1)-Pt(1)-Cl(1) of 176.70 (12)° and 178.20 (5)°, respectively. The average Pt-N and Pt-P bond lengths of 2.040 (4) and 2.1999 (13) Å, respectively are in the range expected for iminophosphane platinum(II) complexes, Ankersmit *et al.*,1996. The torsion angle Pt-P-C(9)-C(8) = -36.5 (4)° indicates that the =CHC_6_H_4_- unit lies below the PtCl_2_(P,N) plane. Selected bond lengths are given in Table 1.

### Experimental

To a dry CH_2_Cl_2_ (10 ml) solution of the precursor [Pt(COD)Cl_2_] was added an equimolar amount of (2(diphenylphosphanyl) benzylidene) (phenyl)methanamine in CH_2_Cl_2_ (10 ml) solution, and the reaction was stirred at room temperature for 1 hr. The yellow solution was concentrated under reduced pressure to half volume and the addition of *ca* 10 ml hexane caused precipitation of the complex, which was filtered off, washed with Et_2_O and dried under vacuum for 4 hrs. Yellow crystals used in the X-ray diffraction studies were grown by slow evaporation of a solution of the compound in a CH_2_Cl_2_-hexane solution at room temperature.

### Refinement

The methyl, methine and aromatic H atoms were placed in geometrically idealized positions and constrained to ride on their parent atoms, with C—H = 0.95 Å for aromatic, C—H = 0.99 Å for ^i^Pr CH, C—H = 0.95 Å for CH and C—H = 0.98 for Me groups.

### Crystal data

**Table:**

[PtCl_2_(C_25_H_21_N_2_P)]	*Z* = 2
*M**_r_* = 646.40	*F*(000) = 624
Triclinic, *P*1	*D*_x_ = 1.878 Mg m^−^^3^
Hall symbol: -P 1	Mo *K*α radiation, λ = 0.71073 Å
*a* = 9.9684 (14) Å	Cell parameters from 16090 reflections
*b* = 10.4129 (15) Å	θ = 2.2–27.1°
*c* = 12.526 (3) Å	µ = 6.46 mm^−^^1^
α = 97.687 (5)°	*T* = 173 K
β = 98.363 (5)°	Block, colourless
γ = 114.499 (3)°	0.07 × 0.06 × 0.04 mm
*V* = 1143.1 (4) Å^3^	

### Data collection

**Table:**

Bruker Kappa DUO APEXII diffractometer	4994 independent reflections
Radiation source: fine-focus sealed tube	4177 reflections with *I* > 2σ(*I*)
graphite	*R*_int_ = 0.058
0.5° φ scans and ω	θ_max_ = 27.1°, θ_min_ = 2.2°
Absorption correction: multi-scan (*SADABS*; Bruker, 2007)	*h* = −12→7
*T*_min_ = 0.671, *T*_max_ = 0.802	*k* = −11→13
16090 measured reflections	*l* = −16→16

### Refinement

**Table:**

Refinement on *F*^2^	Primary atom site location: structure-invariant direct methods
Least-squares matrix: full	Secondary atom site location: difference Fourier map
*R*[*F*^2^ > 2σ(*F*^2^)] = 0.032	Hydrogen site location: inferred from neighbouring sites
*wR*(*F*^2^) = 0.062	H-atom parameters constrained
*S* = 1.01	*w* = 1/[σ^2^(*F*_o_^2^) + (0.0217*P*)^2^] where *P* = (*F*_o_^2^ + 2*F*_c_^2^)/3
4994 reflections	(Δ/σ)_max_ = 0.001
280 parameters	Δρ_max_ = 0.87 e Å^−^^3^
0 restraints	Δρ_min_ = −1.02 e Å^−^^3^

### Special details

**Table:**

Experimental. Half sphere of data collected using SAINT strategy (Bruker, 2006). Crystal to detector distance = 50mm; combination of φ and ω scans of 0.5°, 10s per °, 2 iterations.
Geometry. All esds (except the esd in the dihedral angle between two l.s. planes) are estimated using the full covariance matrix. The cell esds are taken into account individually in the estimation of esds in distances, angles and torsion angles; correlations between esds in cell parameters are only used when they are defined by crystal symmetry. An approximate (isotropic) treatment of cell esds is used for estimating esds involving l.s. planes.
Refinement. Refinement of F^2^ against ALL reflections. The weighted R-factor wR and goodness of fit S are based on F^2^, conventional R-factors R are based on F, with F set to zero for negative F^2^. The threshold expression of F^2^ > 2sigma(F^2^) is used only for calculating R-factors(gt) etc. and is not relevant to the choice of reflections for refinement. R-factors based on F^2^ are statistically about twice as large as those based on F, and R- factors based on ALL data will be even larger.

### Fractional atomic coordinates and isotropic or equivalent isotropic displacement parameters (Å^2^)

**Table:**

	*x*	*y*	*z*	*U*_iso_*/*U*_eq_	
Pt1	0.30212 (2)	0.26883 (2)	0.257890 (16)	0.02056 (6)	
Cl1	0.43855 (18)	0.18884 (16)	0.38298 (11)	0.0407 (4)	
Cl2	0.28642 (15)	0.09332 (13)	0.11921 (11)	0.0312 (3)	
P1	0.18282 (14)	0.34845 (13)	0.14220 (10)	0.0191 (3)	
N1	0.3050 (4)	0.4171 (4)	0.3836 (3)	0.0250 (9)	
N2	−0.1190 (6)	0.2951 (6)	0.5265 (5)	0.0499 (14)	
C1	0.1057 (6)	0.2892 (6)	0.4810 (4)	0.0285 (12)	
C2	0.0286 (7)	0.3573 (6)	0.5293 (5)	0.0407 (15)	
H2	0.0854	0.4555	0.5671	0.049*	
C3	−0.1960 (7)	0.1592 (7)	0.4729 (5)	0.0488 (17)	
H3	−0.3016	0.1133	0.4693	0.059*	
C4	−0.1333 (7)	0.0797 (6)	0.4219 (5)	0.0499 (17)	
H4	−0.1937	−0.0186	0.3854	0.060*	
C5	0.0210 (7)	0.1468 (6)	0.4251 (5)	0.0445 (16)	
H5	0.0675	0.0955	0.3893	0.053*	
C6	0.2735 (6)	0.3698 (6)	0.4900 (4)	0.0289 (12)	
H7A	0.3221	0.3067	0.5062	0.035*	
H7B	0.3173	0.4557	0.5517	0.035*	
C7	0.3209 (6)	0.5449 (5)	0.3819 (4)	0.0273 (12)	
H7	0.3236	0.6003	0.4496	0.033*	
C8	0.3357 (5)	0.6170 (5)	0.2876 (4)	0.0212 (11)	
C9	0.2770 (5)	0.5450 (5)	0.1774 (4)	0.0212 (11)	
C10	0.2852 (6)	0.6262 (5)	0.0963 (4)	0.0276 (12)	
H10	0.2437	0.5783	0.0209	0.033*	
C11	0.3523 (6)	0.7740 (5)	0.1242 (4)	0.0289 (12)	
H11	0.3602	0.8277	0.0678	0.035*	
C12	0.4088 (6)	0.8461 (5)	0.2341 (4)	0.0299 (12)	
H12	0.4527	0.9487	0.2529	0.036*	
C13	0.4012 (6)	0.7687 (5)	0.3156 (4)	0.0273 (12)	
H13	0.4401	0.8178	0.3910	0.033*	
C14	0.1730 (6)	0.3018 (5)	−0.0046 (4)	0.0214 (11)	
C15	0.3084 (6)	0.3303 (5)	−0.0391 (4)	0.0290 (12)	
H15	0.4012	0.3665	0.0138	0.035*	
C16	0.3057 (7)	0.3050 (6)	−0.1516 (5)	0.0365 (14)	
H16	0.3972	0.3256	−0.1755	0.044*	
C17	0.1718 (7)	0.2506 (6)	−0.2277 (5)	0.0404 (15)	
H17	0.1715	0.2347	−0.3042	0.049*	
C18	0.0362 (7)	0.2182 (6)	−0.1947 (5)	0.0386 (14)	
H18	−0.0570	0.1768	−0.2478	0.046*	
C19	0.0400 (6)	0.2477 (5)	−0.0823 (4)	0.0289 (12)	
H19	−0.0514	0.2300	−0.0590	0.035*	
C20	−0.0091 (5)	0.2964 (5)	0.1564 (4)	0.0217 (11)	
C21	−0.0639 (6)	0.3960 (5)	0.1868 (4)	0.0291 (12)	
H21	0.0001	0.4968	0.2001	0.035*	
C22	−0.2132 (6)	0.3464 (6)	0.1973 (5)	0.0371 (14)	
H22	−0.2504	0.4143	0.2184	0.045*	
C23	−0.3071 (6)	0.2025 (6)	0.1780 (5)	0.0387 (14)	
H23	−0.4089	0.1711	0.1851	0.046*	
C24	−0.2551 (6)	0.1018 (6)	0.1481 (5)	0.0358 (14)	
H24	−0.3214	0.0016	0.1340	0.043*	
C25	−0.1056 (6)	0.1471 (5)	0.1384 (4)	0.0304 (12)	
H25	−0.0689	0.0781	0.1199	0.036*	

### Atomic displacement parameters (Å^2^)

**Table:**

	*U*^11^	*U*^22^	*U*^33^	*U*^12^	*U*^13^	*U*^23^
Pt1	0.02045 (11)	0.02191 (10)	0.01956 (11)	0.01090 (8)	0.00208 (8)	0.00264 (7)
Cl1	0.0548 (10)	0.0505 (9)	0.0257 (7)	0.0386 (8)	−0.0057 (7)	0.0005 (7)
Cl2	0.0376 (8)	0.0252 (6)	0.0298 (7)	0.0181 (6)	−0.0007 (6)	−0.0025 (6)
P1	0.0175 (7)	0.0197 (6)	0.0200 (7)	0.0081 (5)	0.0040 (6)	0.0044 (5)
N1	0.020 (2)	0.029 (2)	0.025 (2)	0.011 (2)	0.002 (2)	0.0067 (19)
N2	0.044 (3)	0.048 (3)	0.064 (4)	0.025 (3)	0.020 (3)	0.010 (3)
C1	0.043 (3)	0.033 (3)	0.015 (3)	0.020 (3)	0.010 (3)	0.014 (2)
C2	0.054 (4)	0.038 (3)	0.037 (3)	0.027 (3)	0.012 (3)	0.006 (3)
C3	0.036 (4)	0.062 (4)	0.057 (4)	0.025 (3)	0.021 (3)	0.021 (4)
C4	0.049 (4)	0.036 (3)	0.057 (4)	0.010 (3)	0.021 (4)	0.004 (3)
C5	0.048 (4)	0.037 (3)	0.049 (4)	0.018 (3)	0.020 (3)	0.001 (3)
C6	0.033 (3)	0.036 (3)	0.024 (3)	0.019 (3)	0.009 (3)	0.011 (2)
C7	0.027 (3)	0.026 (3)	0.024 (3)	0.010 (2)	0.004 (2)	−0.001 (2)
C8	0.014 (3)	0.029 (3)	0.019 (3)	0.010 (2)	0.003 (2)	0.005 (2)
C9	0.014 (3)	0.022 (2)	0.025 (3)	0.007 (2)	0.003 (2)	0.001 (2)
C10	0.029 (3)	0.029 (3)	0.025 (3)	0.013 (2)	0.006 (2)	0.008 (2)
C11	0.027 (3)	0.030 (3)	0.032 (3)	0.013 (2)	0.008 (3)	0.015 (2)
C12	0.031 (3)	0.019 (3)	0.036 (3)	0.009 (2)	0.007 (3)	0.004 (2)
C13	0.030 (3)	0.021 (3)	0.030 (3)	0.008 (2)	0.014 (3)	0.005 (2)
C14	0.028 (3)	0.021 (2)	0.019 (3)	0.014 (2)	0.006 (2)	0.006 (2)
C15	0.029 (3)	0.037 (3)	0.024 (3)	0.016 (3)	0.009 (3)	0.008 (2)
C16	0.040 (4)	0.045 (3)	0.036 (3)	0.026 (3)	0.017 (3)	0.013 (3)
C17	0.068 (5)	0.046 (3)	0.020 (3)	0.036 (3)	0.013 (3)	0.006 (3)
C18	0.046 (4)	0.039 (3)	0.023 (3)	0.017 (3)	−0.008 (3)	−0.001 (3)
C19	0.025 (3)	0.037 (3)	0.022 (3)	0.014 (3)	−0.001 (2)	0.002 (2)
C20	0.023 (3)	0.025 (3)	0.016 (3)	0.011 (2)	0.003 (2)	0.004 (2)
C21	0.032 (3)	0.027 (3)	0.029 (3)	0.013 (2)	0.009 (3)	0.006 (2)
C22	0.025 (3)	0.052 (4)	0.041 (4)	0.023 (3)	0.013 (3)	0.007 (3)
C23	0.023 (3)	0.053 (4)	0.037 (4)	0.012 (3)	0.011 (3)	0.012 (3)
C24	0.026 (3)	0.033 (3)	0.045 (4)	0.007 (3)	0.011 (3)	0.018 (3)
C25	0.025 (3)	0.027 (3)	0.036 (3)	0.010 (2)	0.006 (3)	0.006 (2)

### Geometric parameters (Å, °)

**Table:**

Pt1—N1	2.040 (4)	C10—H10	0.9500
Pt1—P1	2.1999 (13)	C11—C12	1.386 (7)
Pt1—Cl2	2.2840 (12)	C11—H11	0.9500
Pt1—Cl1	2.3806 (14)	C12—C13	1.375 (7)
P1—C20	1.803 (5)	C12—H12	0.9500
P1—C14	1.815 (5)	C13—H13	0.9500
P1—C9	1.819 (5)	C14—C19	1.372 (7)
N1—C7	1.276 (6)	C14—C15	1.404 (6)
N1—C6	1.512 (6)	C15—C16	1.392 (7)
N2—C3	1.318 (8)	C15—H15	0.9500
N2—C2	1.332 (7)	C16—C17	1.367 (8)
C1—C5	1.384 (7)	C16—H16	0.9500
C1—C2	1.390 (7)	C17—C18	1.391 (8)
C1—C6	1.507 (7)	C17—H17	0.9500
C2—H2	0.9500	C18—C19	1.392 (7)
C3—C4	1.374 (8)	C18—H18	0.9500
C3—H3	0.9500	C19—H19	0.9500
C4—C5	1.392 (8)	C20—C21	1.393 (6)
C4—H4	0.9500	C20—C25	1.414 (7)
C5—H5	0.9500	C21—C22	1.392 (7)
C6—H7A	0.9900	C21—H21	0.9500
C6—H7B	0.9900	C22—C23	1.362 (8)
C7—C8	1.475 (7)	C22—H22	0.9500
C7—H7	0.9500	C23—C24	1.383 (7)
C8—C9	1.390 (6)	C23—H23	0.9500
C8—C13	1.405 (6)	C24—C25	1.393 (7)
C9—C10	1.396 (7)	C24—H24	0.9500
C10—C11	1.369 (7)	C25—H25	0.9500
			
N1—Pt1—P1	88.47 (12)	C11—C10—H10	119.6
N1—Pt1—Cl2	176.70 (12)	C9—C10—H10	119.6
P1—Pt1—Cl2	91.76 (5)	C10—C11—C12	120.5 (5)
N1—Pt1—Cl1	91.27 (12)	C10—C11—H11	119.7
P1—Pt1—Cl1	178.20 (5)	C12—C11—H11	119.7
Cl2—Pt1—Cl1	88.60 (5)	C13—C12—C11	119.8 (5)
C20—P1—C14	106.2 (2)	C13—C12—H12	120.1
C20—P1—C9	106.1 (2)	C11—C12—H12	120.1
C14—P1—C9	104.7 (2)	C12—C13—C8	120.0 (5)
C20—P1—Pt1	111.62 (16)	C12—C13—H13	120.0
C14—P1—Pt1	119.12 (15)	C8—C13—H13	120.0
C9—P1—Pt1	108.20 (17)	C19—C14—C15	119.4 (5)
C7—N1—C6	114.4 (4)	C19—C14—P1	122.4 (4)
C7—N1—Pt1	127.8 (4)	C15—C14—P1	118.2 (4)
C6—N1—Pt1	117.6 (3)	C16—C15—C14	119.4 (5)
C3—N2—C2	116.7 (5)	C16—C15—H15	120.3
C5—C1—C2	116.9 (5)	C14—C15—H15	120.3
C5—C1—C6	122.7 (5)	C17—C16—C15	120.3 (5)
C2—C1—C6	120.4 (5)	C17—C16—H16	119.9
N2—C2—C1	124.8 (6)	C15—C16—H16	119.9
N2—C2—H2	117.6	C16—C17—C18	121.0 (5)
C1—C2—H2	117.6	C16—C17—H17	119.5
N2—C3—C4	124.2 (6)	C18—C17—H17	119.5
N2—C3—H3	117.9	C17—C18—C19	118.6 (5)
C4—C3—H3	117.9	C17—C18—H18	120.7
C3—C4—C5	118.3 (6)	C19—C18—H18	120.7
C3—C4—H4	120.8	C14—C19—C18	121.3 (5)
C5—C4—H4	120.8	C14—C19—H19	119.3
C1—C5—C4	119.1 (5)	C18—C19—H19	119.3
C1—C5—H5	120.4	C21—C20—C25	119.4 (5)
C4—C5—H5	120.4	C21—C20—P1	123.1 (4)
C1—C6—N1	110.5 (4)	C25—C20—P1	117.5 (3)
C1—C6—H7A	109.6	C22—C21—C20	119.4 (5)
N1—C6—H7A	109.6	C22—C21—H21	120.3
C1—C6—H7B	109.6	C20—C21—H21	120.3
N1—C6—H7B	109.6	C23—C22—C21	121.3 (5)
H7A—C6—H7B	108.1	C23—C22—H22	119.3
N1—C7—C8	127.9 (4)	C21—C22—H22	119.3
N1—C7—H7	116.1	C22—C23—C24	120.3 (5)
C8—C7—H7	116.1	C22—C23—H23	119.8
C9—C8—C13	120.0 (5)	C24—C23—H23	119.8
C9—C8—C7	124.4 (4)	C23—C24—C25	120.2 (5)
C13—C8—C7	115.3 (4)	C23—C24—H24	119.9
C8—C9—C10	118.8 (4)	C25—C24—H24	119.9
C8—C9—P1	119.8 (4)	C24—C25—C20	119.5 (4)
C10—C9—P1	121.4 (4)	C24—C25—H25	120.3
C11—C10—C9	120.8 (5)	C20—C25—H25	120.3
			
N1—Pt1—P1—C20	−71.2 (2)	C8—C9—C10—C11	1.2 (7)
Cl2—Pt1—P1—C20	105.47 (17)	P1—C9—C10—C11	178.0 (4)
N1—Pt1—P1—C14	164.4 (2)	C9—C10—C11—C12	−2.3 (8)
Cl2—Pt1—P1—C14	−18.93 (19)	C10—C11—C12—C13	1.8 (8)
N1—Pt1—P1—C9	45.12 (19)	C11—C12—C13—C8	−0.2 (7)
Cl2—Pt1—P1—C9	−138.17 (16)	C9—C8—C13—C12	−0.8 (7)
P1—Pt1—N1—C7	−36.2 (4)	C7—C8—C13—C12	−174.6 (4)
Cl1—Pt1—N1—C7	142.0 (4)	C20—P1—C14—C19	4.1 (5)
P1—Pt1—N1—C6	138.9 (3)	C9—P1—C14—C19	−107.9 (4)
Cl1—Pt1—N1—C6	−42.8 (3)	Pt1—P1—C14—C19	131.1 (4)
C3—N2—C2—C1	−0.5 (9)	C20—P1—C14—C15	−179.6 (4)
C5—C1—C2—N2	0.8 (9)	C9—P1—C14—C15	68.4 (4)
C6—C1—C2—N2	−179.5 (5)	Pt1—P1—C14—C15	−52.6 (4)
C2—N2—C3—C4	0.7 (10)	C19—C14—C15—C16	0.9 (7)
N2—C3—C4—C5	−1.1 (10)	P1—C14—C15—C16	−175.6 (4)
C2—C1—C5—C4	−1.1 (8)	C14—C15—C16—C17	−1.1 (8)
C6—C1—C5—C4	179.2 (5)	C15—C16—C17—C18	−0.6 (8)
C3—C4—C5—C1	1.3 (9)	C16—C17—C18—C19	2.5 (8)
C5—C1—C6—N1	77.7 (6)	C15—C14—C19—C18	1.1 (7)
C2—C1—C6—N1	−102.0 (5)	P1—C14—C19—C18	177.4 (4)
C7—N1—C6—C1	93.8 (5)	C17—C18—C19—C14	−2.8 (8)
Pt1—N1—C6—C1	−82.0 (4)	C14—P1—C20—C21	−109.9 (4)
C6—N1—C7—C8	−172.7 (5)	C9—P1—C20—C21	1.2 (5)
Pt1—N1—C7—C8	2.5 (8)	Pt1—P1—C20—C21	118.8 (4)
N1—C7—C8—C9	26.9 (8)	C14—P1—C20—C25	71.8 (4)
N1—C7—C8—C13	−159.6 (5)	C9—P1—C20—C25	−177.1 (4)
C13—C8—C9—C10	0.3 (7)	Pt1—P1—C20—C25	−59.5 (4)
C7—C8—C9—C10	173.6 (4)	C25—C20—C21—C22	−0.9 (8)
C13—C8—C9—P1	−176.5 (3)	P1—C20—C21—C22	−179.1 (4)
C7—C8—C9—P1	−3.3 (6)	C20—C21—C22—C23	−0.4 (8)
C20—P1—C9—C8	83.4 (4)	C21—C22—C23—C24	0.5 (9)
C14—P1—C9—C8	−164.5 (4)	C22—C23—C24—C25	0.6 (9)
Pt1—P1—C9—C8	−36.5 (4)	C23—C24—C25—C20	−1.8 (8)
C20—P1—C9—C10	−93.4 (4)	C21—C20—C25—C24	1.9 (8)
C14—P1—C9—C10	18.7 (4)	P1—C20—C25—C24	−179.7 (4)
Pt1—P1—C9—C10	146.7 (4)		
